# The Role of Health Kiosks in 2009: Literature and Informant Review

**DOI:** 10.3390/ijerph6061818

**Published:** 2009-06-11

**Authors:** Ray Jones

**Affiliations:** Faculty of Health and Social Work, University of Plymouth, Plymouth PL4 8AA, UK; E-Mail: ray.jones@plymouth.ac.uk; Tel.: +44 1752 586532

**Keywords:** kiosk, health systems, Internet

## Abstract

Kiosks can provide patients with access to health systems in public locations, but with increasing home Internet access their usefulness is questioned. A literature and informant review identified kiosks used for taking medical histories, health promotion, self assessment, consumer feedback, patient registration, patient access to records, and remote consultations. Sited correctly with good interfaces, kiosks can be used by all demographics but many ‘projects’ have failed to become routine practice. A role remains for: (a) integrated kiosks as part of patient ‘flow’, (b) opportunistic kiosks to catch people’s attention. Both require clear ‘ownership’ to succeed.

## 1. Introduction

**Figure 1.**
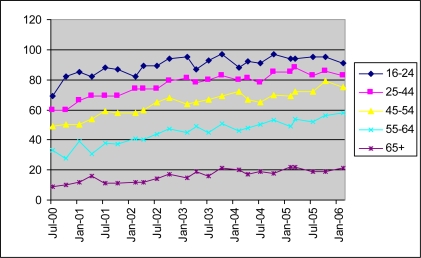
Percentage of different age groups in Great Britain who have ever used the Internet (constructed from NOS statistics) [8].

Many have expressed concerns about equitable access to the Internet (e.g. [1–5]). For example, although home Internet access for the U.K. as a whole had increased from just over 30% in 2000 to 55% in 2005 and to 65% by 2008, there is still variation by income, region and mainly by age [6,7]. In 2006 in the U.K., 87% of 16–30 year olds had used a computer in the previous three months compared with 45% of those aged 50 and over [8] (Figure 1). Information can be physically available through many different private, social, or public sources. Information is available through private sources such as TV, home Internet or telephone. Socially, information may be available onscreen or on paper through a family member with Internet access. Public sources include kiosks and public libraries providing Internet access.

NHS Choices is an organisation within the English National Health Service (NHS) responsible for the NHS Choices portal (www.nhs.uk) to information and services. They were concerned about equity of access to their information, but had divided opinion about kiosk use. On the one hand, some thought kiosks were a way of addressing the ‘digital divide’, whereas others remembered that the NHS kiosk had not been particularly successful and had been withdrawn. They commissioned a review. The full report was submitted to NHS Choices in February 2008. This paper is an updated summary of that report.

**Figure 2.**
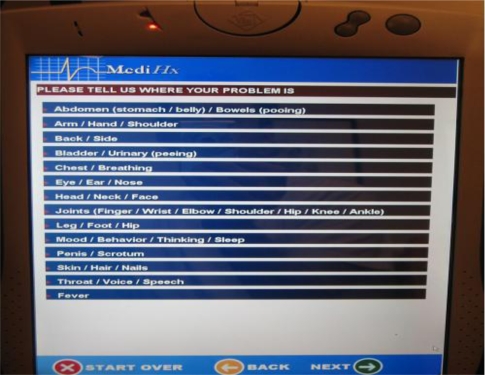
‘First generation’ tablet computer used for patient interviewing in an emergency department in Ontario, Canada [12].

The term kiosk tends to be used for public access touch screen computer that is normally used standing up. Some applications have been delivered in booths – i.e. a touch screen computer not necessarily in such a robust casing but used while seated (e.g. [9,10]). Other applications have simply used desktop computers possibly with touch screen, or rollerball (e.g.[11]), or just a mouse. Other studies have used various types of tablet or laptop (Figure 2) (e.g. [12,13]).

The main feature of all approaches is public access computing with the specific aim to give or collect information, with special attention to ensure accessibility. This review included all public access computing.

## 2. Methods and Data Sources

Web of Knowledge (WOK), Medline, and Google Scholar were searched using keywords including *kiosk* or *touchscreen* and by citation and author searching on WOK. Informants were identified from personal knowledge, from the literature, by ‘snowballing’, and by contacting (February 2008) two discussion lists (Patient Information Forum, with over 700 members, and Consumer-Health-Informatics, with around 200 members) asking for ‘updates’ on current kiosk use, and by web searches. The report also made use of the author’s own publications and unpublished work and two previous reviews of kiosks: (i) Boudioni [14] produced a summary in 2003 commissioned by the Access, Booking and Choice Directorate of the NHS, and (ii) Nicholas *et al.* produced numerous publications summarised in 2004 by a ‘sourcebook’ of their work [15]. Over 220 individual emails were sent to 104 people in addition to telephone and face-face enquiries. The literature review and personal contacts identified 229 publications including published papers, grey literature and unpublished reports. The preliminary results were presented and discussed in an online interactive webinar attended by 65 people internationally on 26/3/2008. (The recording is available on the Internet [16]). The 61 page report has been summarised and updated in this paper.

## 3. Results and Discussion

### 3.1. Opportunistic Versus Integrated into Clinical Process

Kiosk use can be classified as (a) *Opportunistic kiosks* that are placed in locations and wait for opportunistic use, (b) *Integrated kiosks* that have been designed into the clinical process. Table 1 gives examples of publications between 2002–2007.

### 3.2. Opportunistic Kiosks

#### Consumer health information, health education and promotion:

People want information about their health and will seek it (*information pull; consumer health information*). In the U.K., there has been a flourishing trade in lay health care guides since the Middle Ages [17,18] illustrated in more recent decades by problem pages [19], telephone help lines, leaflets and booklets and later websites [20]. The Internet is clearly a major resource for *health information ‘pull’* [21,22]. On the other hand, professionals want to change patients’ knowledge and attitudes and *push* information to try to achieve this. The need for health promotion and patient education can be traced through awareness of tuberculosis as a leading cause of death in the 1800s [23] to 1960s studies linking smoking and lung cancer [24]. Health promotion originated from a Canadian government report in 1974 [25] and in the U.K. has mostly been through regional and national initiatives using television, radio, billboards, leaflets, health fairs and other methods to convey healthy lifestyle messages [26]. Patient education was recognised from the 1960s [27] as an important part of chronic disease management. Researchers started using computers for patient education in the mid-80s e.g., a computer-printed paper based feedback system for informing diabetes patients [28] and a simulation of a dialysis unit to train renal patients [29]. While TV and the mass media have a clear role in health promotion (information push), and (use of drama and ‘personality stories’ may be more effective in promoting change of attitudes or behaviour than direct advertising [30]), the role of the Internet in *information push* is less clear. Patients have to be motivated to find and use an Internet computer to seek this information, thus having already become information seekers.

#### Early kiosk use:

**Figure 3.**
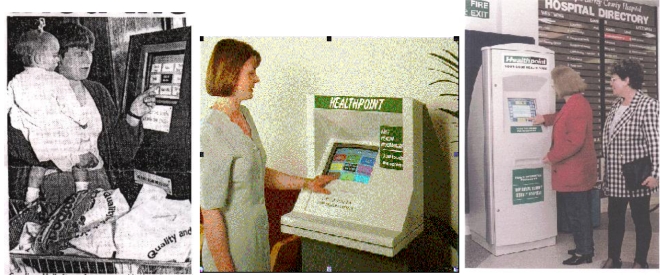
Healthpoint in (from left) Maryhill Shopping Centre Glasgow 1991, 1994, and in a Surrey Hospital 1996.

An early example was Healthpoint (Figure 3), a community based touch-screen kiosk developed and evaluated in Glasgow in 1989 [31]. In the early 1990s, Healthpoint kiosks were sited in supermarkets, shopping centres, community pharmacies, health centres, hospitals, bars, sports centres, post offices, job centres, and libraries, amongst the 23 sites tried [32]. Information provided included both public health and lifestyle topics (smoking, alcohol, sex, drugs, stress) as well as more condition specific information such as prostate cancer. There were few problems in finding locations to site kiosks [33]. In 1992 these kiosks were used during five months by seventeen percent of a random population sample. The prevalence of users amongst the over 50s (13%) was not much less than the 20% of users aged under 50. Users were observed in bars using kiosks in groups to access information such as sexually transmitted diseases, smoking, and alcohol use. The most popular topics varied by site but were always public health themes. Medical dictionary and condition specific topics were rarely accessed. Interview data showed a more positive reaction from ‘less educated’ than educated people and was expected because of the style of presentation. In a subsequent 1996 study, nine percent of people who had used a kiosk in one sports centre had apparently obtained no other form of health information in the previous two weeks [34]. Various ‘Healthpoints’ for particular patient groups were also trialled in outpatient areas, for example in radiology [35].

**Figure 4.**
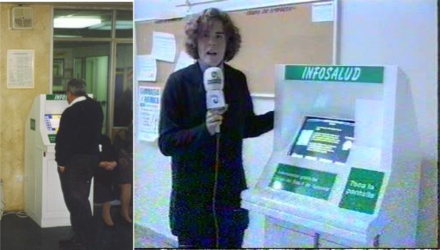
Infosalud in (from left) Segovia General Hospital and on Segovia TV, 1999.

Kiosks were used in Andalucia in Spain as part of a system of registration using a “tarjeta sanitaria” (health card) since the late 1990s. The Healthpoint system was translated into Spanish and trialled as Infosalud (see Figure 4) in 1999 [36]. The European project, Tesemed [37,38], provided information about over-the-counter medications and also made use of general health information from Infosalud.

#### NHS kiosk:

**Figure 5.**
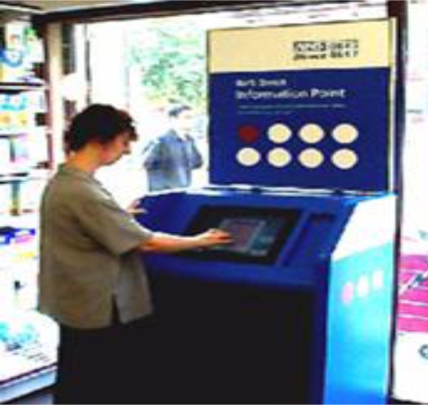
NHS Kiosk, 2001.

To improve access to both health information and health services in England, the NHS Direct telephone helpline was established in 1998 followed in 1999 by the NHS Direct Online Web site. Aiming to improve equity of access to web site information, the first NHS kiosk was installed in September 2000, 81 were installed by February 2001, and 136 by October 2001. Kiosks were designed in ‘NHS blue’ and had a ‘corporate appearance’ (Figure 5). Information presented was similar to that on the website. Locations included NHS ‘walk-in centres’, community centres, retail pharmacists, hospitals, public libraries and retail sites. Nicholas *et al.* studied both NHS and In Touch with Health kiosks resulting in numerous publications including [15,58,59,61,65,67–74].

Jones [75] carried out a study of NHS kiosks in 2001. Automatic monitoring statistics were produced for all 136 kiosks for four months showing number of user episodes. A sample of twenty kiosks representative of type of site and geographical location was taken for an ‘exit poll’ and geographically defined postal survey. A total of 1,666 people were interviewed leaving nineteen sites. (One site was unable to participate in interviews.) Postal questionnaires were sent to 1,400 randomly selected households living within five kilometres of the twenty kiosks. Kiosk sites and respondents to the postal survey were classified by deprivation category. All 1,652,586 English postcodes were ranked according to an index of multiple deprivation and postcode areas then classified according to their decile of deprivation.

Routine statistics for all 136 kiosks showed they were used on average twelve times a day; one was used 49 times but a fifth less than four times a day. Fifty-three percent of kiosks were in the two most deprived but eight percent were in the four most affluent deciles of English postcodes. Leisure centres, tourist sites, hospitals, and supermarkets had the highest usage and community and education sites the least use. As a result of the opening hours of each site, kiosks were available from 20 hours a week through to 168 hours (24/7) a week and (not surprisingly) those available for longer hours were used more. A third of those interviewed leaving the site had seen the kiosk but only 6% (94) had used it, the main stated reason for non use being that they did not know what it was. Only nine gave their reason for not using the kiosk as their ability to get information from the Internet. Overall 63% might use it in the future but this varied from 2% to 100% by site. Older people were less likely to have noticed the kiosk, used it or possibly would use it in the future.

Those who had obtained other health information were more likely to use the kiosk than people who had not obtained other health information (8% vs 4%; χ^2^ = 7.4; 1df; p = 0.006). The kiosk attracted users who were already seeking information from computers, written information, or who had used NHS Direct in the last two weeks. A small minority (39, 2% of total) used the kiosk who had not obtained any other health information in the last two weeks. These 39 were younger (37 vs 46 years old; t = −3; 1642df; p = 0.002) but there was no difference in gender, car ownership, and first language with the rest of the interviewees. Thirty-four (87%) said they were likely to use the kiosk again.

Just under half (44%) of the postal respondents had obtained health information from any source in the last two weeks, the majority (33%) being in face-to-face contact. Fifty-eight percent (160) of those with home or work access to the Internet had used it to obtain health information at some time. Nineteen had used the Internet without access at home or work. Of the 223 who had Internet access but had not used it, 127 (57%) said they may use the touch-screen kiosk. A quarter had obtained health information from the Internet, 36% had not used the Internet to obtain health information nor would they use the kiosk, but 39% who had not used the Internet would use the kiosk now that they knew about it.

Although the study provided some evidence that kiosks can increase accessibility of health information, level of use was low. Many kiosks could have been better sited and this study was used to review locations. Sites such as supermarkets and leisure centres, visited frequently by people aged over 60, were more likely to reach those who did not use the Internet for health information. Many did not notice the kiosk and others did not know what it was. A less ‘corporate’ kiosk design may have been more eye-catching and may have encouraged more users. NHS kiosks were decommissioned around 2005 (Bob Gann, personal communication).

#### Recent uses in health education:

Kiosks continue to be used with many recent studies being reported from the U.S.A. Rather than trying to cover the whole range of health information most kiosks, sited in community or health service settings gave information with specific educational aims, for example, to promote child health [41] or give general health information [15,47,54,57,60,76], improve use of antibiotics for respiratory infections [40], encourage uptake of breast cancer screening [45], address needs of ethnic minorities [48], help with diabetes management [77], provide information in outpatient areas [55,56], provide individuals with their risk of cancer [78,79]; manage different types of headaches [80], teach safe sex negotiation skills to adolescents [81], educate about skin cancer [11,82], assess food safety knowledge in schools [83], improve tuberculosis management [84], encourage weight loss [85], and promote healthy eating or better nutrition [62,86,87]. Some kiosks (e.g. Wellpoint) include blood pressure, body fat and body mass index measurement as well as giving health information. Wellpoint, for example, has been installed in many occupational health settings [88].

#### Patient access to records:

The drivers towards giving patients access to their own computer-held medical records have included the desire for more patient involvement in chronic disease management, aims to improve the collection of clinical data through patient interviewing, but also ethical considerations and concerns for patient empowerment. Some U.K. G.P.s, such as Brian Fisher have routinely given patients access to their paper record for two decades [89,90]. The ‘cause’ has been helped by the push from legislation and initiatives such as the Copying Letters to Patients [91]. Early studies of computer access included touchscreen access to records in a Glasgow general practice [10] and later access to secondary care records in randomised trials in cancer [9] and schizophrenia [92] using touch screen booths. More recently, Pyper *et al.* [93–95] explored patient access to their online records in an Oxfordshire general practice with promising results but the system failed to be adopted as routine practice. In renal medicine, a specialty that has always been at the forefront of clinical computing, patients via Renal Patient View can have access to their records via the web although as yet no major trial has assessed its impact [96]. Most of these studies have been of opportunistic use or as an ‘optional extra’ to routine clinical care.

### 3.3. Kiosks Built into Clinical Process

#### Computer-patient interviews:

Computers have been used for patient interviews for over 40 years; with Slack *et al.* pioneering this use in 1966 [97]. During the 1990s the NHS Information Management Group commissioned reviews [98] and workshops on direct patient entry to the computer, realising that computer-taken patient histories could make the consultation more effective and efficient and provide a partial solution to the data collection problem of clinical records. Some systems also offered tentative diagnoses e.g. [99]. Certain specialties and health problems, such as mental health and back pain, were advanced in this approach, even before the Internet started to be widely used. However, few of these systems became integrated into routine care. On the other hand, it was clear that patient interviewing could be combined with education, health promotion, and possibly data input from various physical examinations in a ‘patient workstation’ [100]. More recent publications describe use in emergency departments to assist in asthma management [50–53], pre-operative history before anaesthesia [101], emergency walk in inner-city clinic [102], preconsultation use of a computer part-interview part-education in a diabetes clinic [103,104], and assessment of anxiety and depression in cancer patients [66,105]. Computer patient interviewing using both web based applications in ‘office’ applications is starting to become widespread in the USA, using packages such as Instant Medical History [106].

#### Consumer Feedback:

**Figure 6.**
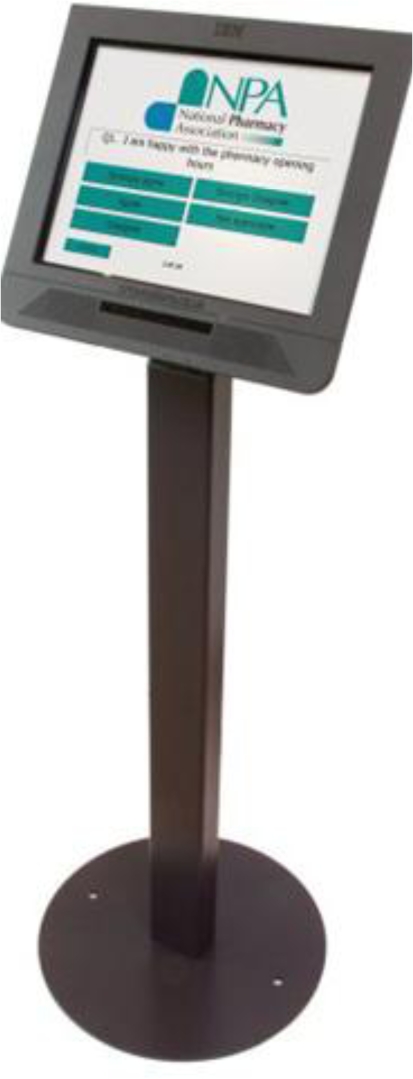
Touchscreen kiosk used for consumer feedback (from http://www.crtsolutions.co.uk/index.php?pageid=15).

Touchscreens have been used for consumer (patient) feedback in a number of sites including an outpatient area in Lothian [49], inpatient setting in hospitals in Slough England [42], and for health professional conference feedback in Fife [107] (Figure 6). It depends on whether their use is prompted or not as to whether they should be classified as opportunistic or built into the clinical process.

#### Patient registration and clinic organisation:

An increasing use is for patient registration and to improve the flow of patients through a clinic or general practice. For example, the EMIS general practice system (which has about half the English market) has offered a patient registration kiosk since 2005 [108]. Other UK examples include hospital outpatient clinics [109] and community clinics [110]. There are also numerous examples from US primary care [111,112].

#### Remote consultation and patient monitoring:

Various videoconference type applications are becoming more routine such as that reported in a 2008 press report of the Scottish trial of teleconsultations in Aberdeen where a patient booth includes stethoscope, blood pressure cuff and thermometer [39].

### 3.4. Longevity

Many of the kiosks reported in the literature never became a routine part of service delivery. Table 2 shows the largest installations that I have identified and if/when they were withdrawn.

### 3.5. Hardware Issues

Two hardware issues are worth describing in more detail as they may have been key to outcomes of kiosk use in some cases.

#### Printers:

In many cases having a printer on publicly sited kiosks has resulted in a high maintenance overhead for local staff and can be a major reason for dissatisfaction with kiosk use (e.g. informant Surrey PCT .... *”the machine we had at our local community hospital in the main reception area .... was eventually removed due to lack of use - misuse - maintenance issues - nightmare trying to refit the paper roll”*). ATMs (cashpoints) of course use printers; these may also have high maintenance but have high utility for bank staff. The breast cancer information kiosk described by Kreuter [45] had as its main purpose the production of a booklet and (although I did not obtain information on local maintenance needed) may have been worth the work. My own studies of cancer information were also based on the production of a booklet [116] – but this was completed by a research assistant and ‘offline’ from the patient’s use of the touchscreen computer as previous experience had suggested inclusion of printers created high maintenance in a public access situation.

#### Handsets:

Some of the NHS kiosks in 2001 had handsets for connection to an NHS Direct operator. Handsets can also be use just locally to allow a user to hear sound from the kiosk. Picking up and replacing the handset can also be used effectively to mark beginning and end of an episode [117]. In 2007 Glasgow ‘reinvented’ kiosks with handsets in a ‘Scottish Info Pod’ [118]. *“The ‘info pods’, the first of their kind in NHS Scotland, are new stand alone electronic information points, designed to provide patients with a range of information <snip>...Gartnavel General Hospital and Easterhouse Health Centre....<snip>.. healthy eating, exercise, stopping smoking and hand hygiene, <snip>..free standing telephone help-point ...<snip>.. local taxi company, Smokeline, Travel Line....”*.

### 3.6. Kiosk Locations

Community sites are normally used for opportunistic kiosks, although (e.g.) public library based kiosks or booths could be used as part of a library referral scheme. Health care settings might be used both for opportunistic kiosk use but more likely for referred or integrated use.

#### Opportunistic community sites:

Nicholas *et al.* [15] based upon their studies of In Touch with health and NHS Direct kiosks concluded that information centres and hospitals had comparatively long session length and reasonably high overall usage. Public places such as supermarkets, did well in so far as they offered a large potential body of users, but use was cursory, and session length relatively short, and kiosks in surgeries performed poorly because of lack of anonymity and time anxiety. This was in broad agreement with my early work with Healthpoint in Glasgow [32–34] and my own study of NHS Direct kiosk in 2001 [75]. Supermarkets and public places seemed to get high usage. Waiting rooms were often not a good site as people were concerned about privacy and concerned about missing their appointment. Kiosks ‘hidden away’ in back rooms of pharmacies were not seen and received hardly any use [75]. However, conclusions about location also have to take into account the style of kiosk and the content presented. Both In Touch with Health and NHS Direct kiosk were ‘corporate’ in appearance and relatively sober in content. Healthpoint in 1993 had a more lighthearted appearance both in the casing and in the content. Healthpoints were well used in some bars by groups of users.

Nicholas *et al.* [15] concluded from their studies of In Touch that: “*People were put off using the kiosk in situations where they could be observed and so lacked privacy. Just under half of non-users (47%) said that they did not like the idea of using a surgery kiosk because it was in a public place. ....’search disclosure’ is thought to impact most strongly on the use of kiosks in surgeries, some hospital waiting room areas and kiosks located in front of a pharmacy or shop queues. Users preferred to use the kiosk in ‘designated information areas’, such as in Information centres, or in such designated areas in surgeries and hospitals, where they cannot be observed, or where use was considered socially acceptable.”* However, I think this over-generalises. The Healthpoint experience was also that the front of a general practice waiting room was not a good position because people do not want to be watched, but for example kiosks in a busy supermarket were used. People would use a kiosk in a group in a pub ‘for a laugh’ but in the quiet of a public library people required more privacy. Colleagues would not leave a group in a staff canteen to use a kiosk (no matter how well shielded) that everyone knew contained health information [119]. Those situations where kiosk/touchscreen use is ‘expected’, i.e. built in to the process of the location such as to register on arrival or to follow up an information prescription on leaving can be ‘seen’ but shielded.

#### Willingness of community sites to host a kiosk:

Kreuter *et al.* in St Louis in 2003–2004 contacted 110 potential kiosk hosts from five different types of community settings [45]. Of these, 44 (40%) agreed to host the kiosk and 41 (37%) actually hosted it. At one of the 41 host sites, a Laundromat, all user data were lost due to a computer malfunction; this left the final study sample of 40 kiosk host sites. Recruitment of Laundromats and beauty salons required the greatest effort. They contacted 37 Laundromats to identify the 7 that agreed to host the kiosk (19% participation) and 28 beauty salons to identify 8 host sites (29%). Participation rates were higher for social service agencies (73%), neighbourhood health centres (67%), and churches (42%). However, usage per day was highest for Laundromats (14/day), followed by neighbourhood health centres (10/day), churches (9/day), social service agencies (9/day), and beauty salons (5/day). In the first phase of Healthpoint studies in 1991–92 the University of Glasgow approached 22 community sites of which 17 agreed [32]. Sites included retail (e.g. Boots the Chemist who cleared shelf space to make space for the kiosk), bars, social security offices, further education, as well as health service sites. Later studies (1996) had similarly high acceptance rates [117]. It seems likely that acceptance rates for large scale non-research non-local implementations is likely to be less. Furthermore, acceptance rates now are likely to be much lower as since the early 1990s organisations have become far more concerned about health and safety, vandalism, liability and legal responsibility.

#### Health service and health related sites:

The main problem with health service settings is lack of space. A study of 269 hospital Emergency Departments in the U.S.A. found that 54% did not have space for a kiosk [120]. A NICE review of computerised cognitive behavioural therapy [121] cited our study of computerised cognitive behavioural therapy for anxiety in which we tried to site booths in health centres in Glasgow as well as public libraries. Public libraries were keen to include booths (a large desk with patient-use while seated) and had space whereas only one out of six large health centres had space (and that space was not very suitable).

Although some studies such as Pyper’s work in Bury Knowle [93] have included booths for patients it is easier in general practice, though still not easy, to site kiosks (i.e. stand to use). However, as described above, kiosks will not be used if the user is overlooked – unless that use is expected. From their studies of In Touch with Health kiosks Nicholas *et al.* concluded *‘Generally little thought appears to have gone into the integration of the health information kiosks into the normal routines of health environments. However, where kiosks were actively promoted by health staff, this integration was shown to impact positively on use, firstly because there was a culture in promoting the kiosk and secondly there were people on hand to help people use the system. Few kiosks were embedded in their location. Health staff have to be made aware of the impact that information systems can have for patients. It may also be useful for such systems to be networked to the surgery consulting rooms themselves, so that doctors can be more pro-active. This may be difficult in regard to time availability, but current practices - often just letting the patients ‘get on with it’ - are hardly acceptable, and lead to under-exploitation of a potentially valuable health aid.” [15].*

### 3.7. Kiosk Users

It is difficult to disentangle the location, the look, the interface, the information offered and the demographics of the users. Furthermore, we have to be careful about the methods used to assess or record the demographics of users. If this is via onscreen questionnaire with no validation, it may not be wholly accurate (a) because ‘episodes of use’ are not always easy to define and (b) for opportunistic information retrieval users may not answer demographic questionnaires accurately [117].

#### Age:

Although I have argued elsewhere that kiosks that look too ‘corporate’ may not get much opportunistic use, some of the early Healthpoints (with cartoon doctors painted on the side) were sited near children’s ‘toys’ in supermarkets and so got only children using them. They had to be moved to a more ‘adult’ area and made to look a bit more ‘serious’. Nicholas *et al.* found that children accounted for a large part of the use of In Touch kiosk and noted that 41% of pages viewed at GP surgeries were accounted for by the under 15s compared to 25% of pages viewed in hospitals because children are much more likely to visit surgeries than hospitals.

Nicholas *et al.* reported some fairly ‘common sense’ findings on older people: that they were less likely to search deeply compared to others, would have shorter sessions, were less likely to find the system very easy to use, less likely to say they were comfortable with the technology etc. However, if the aim is to ensure that older people are not excluded from such health information we need to know how many older people can and will use this technology compared to Internet use. In a population survey of Healthpoint use in 1992, 13% of people over 50 had used a Healthpoint compared to 20% under 50. In 2007, around 80% of the under 50s had used the Internet compared to around 40% of the over 50s. In Touch with Health kiosks were not specifically designed with older people in mind. My personal view is that the interface was rather complicated (compared to (say) Healthpoint) requiring too many ‘touches’ to get to information. (This is the disadvantage of making a kiosk comprehensive at all times). The need for simplicity in kiosk interface is echoed by Worger of Starthere who said (email May 2009) “...it was clear that there was a need for the simplest interface and design if kiosks were to engage typically digitally excluded groups such as the elderly and offenders (i.e. putting a standard website on a kiosk doesn’t do the job).” If the aim of using kiosks is to engage older people the interface needs to be designed with them and for them [122].

#### Social class and deprivation:

People in areas of deprivation and lower social class have less Internet access than those in more affluent areas [8]. Will kiosks be used by people in areas of social deprivation and does their use help lessen the digital divide? NHS kiosks sited in 2001 were fairly well sited by area of deprivation: more than half the kiosks were sited in the 20% most deprived areas in England, however 8% of the kiosks were sited in the 40% most affluent areas. The use of NHS kiosk was limited partly because of poor local siting (e.g. one in a back room of a pharmacy). It was not the aim of the NHS kiosk to be able to compare affluent Vs deprived used and there were too many other variables (e.g. type of site (retail Vs pharmacy Vs health centre) to be able to make such a comparison. Nicholas *et al.* summarising their experiences of kiosk evaluations said “*With regard to kiosk use where a neighbourhood housing a kiosk had a high incidence of mortgages, generally there were a lower number of kiosk users, these users might well have their own Internet access.*” This suggested that at least people in deprived areas were no LESS likely to use kiosks and kiosks might help somewhat in making information more available. But as opportunistic kiosks are used mainly used for a very short time and are best used in information push rather than information pull (seeking), kiosks are probably not a good way to try to reduce digital inequalities.

#### Disabilities:

In her report to the Access, Booking and Choice Directorate of the NHS Boudioni [14] said *“Most of these companies have their own quality criteria or a user group to advise them on design and development, as they realise that users’ needs, experiences and confidence are not uniform...... At least one of these companies has put together approval criteria for disabled access such as access to a touch screen by lying on their sides, sufficient width and depth for the wheelchair, appropriate height and within easy reach screen, size of the screen, type of touch technology and physical stability.... They have also considered access and use of children and elderly people. Elderly people may have specific needs, as one of the developers said: „Somewhere to put their walking stick on, as they commonly like to lean on the touch screen as it is more steady than their walking stick” Other companies also consider disability and access issues, and their screens are tested rigorously to meet specification criteria.”* Boudioni cited a number of company websites in support of this statement including In Touch with Health, Technology Active Solutions, and NeoProducts.

On the other hand Nicholas *et al.* said “Health professionals acknowledged that the In Touch with Health kiosks were difficult for wheelchair-bound people to use as they were designed for operation at a standing position. In Touch with Health recognised this problem themselves, and their ‘new generation’ web-enabled kiosks were all made to be suitable for wheelchair users. NHS kiosks could also be used at seat (i.e. wheelchair) level. Other than this, the kiosk systems evaluated had no provision of any kind for the disabled.”

There has been work on developing computers and kiosks for particular disability groups, such as for the deaf e.g. [123], on kiosks for older people [124]. But probably the biggest disability in having access to health information is lack of English literacy whether due to ‘disability’ such as severe dyslexia, learning disability or poor level of educational attainment, or as a result of immigration or ethnic differences not having English language skills. (See ethnicity, literacy and language below).

#### Ethnicity, literacy and language:

I am not aware of evidence about whether ethnicity per se (i.e. when separated from issues of language) has led to less or more kiosk use. Statistics are not routinely published on Internet access by ethnicity. In my study of NHS kiosks [75] I found little difference in Internet use between those with English as a first language and others. Nicholas *et al.* found that place of birth had an impact on perceived ease of use of In Touch kiosks but this was associated with socio-economic status: users born in the UK who were employed as skilled workers were twice as likely to find kiosks very easy to use compared to non-UK born users and UK born unskilled users. Clearly if kiosks are to offer culturally sensitive information (in English) then, just as kiosk information should be tailored to the local environment so that should take into account ethnic variation within that local environment.

Boudioni stated that *“Touch screens with health related information in other languages have been produced by In Touch with Health <snip>. Information in some ethnic minority languages is available on PALS Bradford kiosks; information in Gujarati, Bengali, Urdu and Chinese is available on Oldham NHS Trust kiosks.”*

The ‘Three cities project’ [125] made information available on 10 health topics, translated into five Languages, available on touch screen in each of Sheffield, Nottingham and Leicester between approximately 2002 and 2005. Kiosks were rotated through a series of locations including a library, GP practice, and a temple. At the end of the project however the information was put onto the web at www.soundshealthy.nhs.uk and by 2008 had had about 20,000 accesses but there was no money for updating, so when the information becomes out of date the site was to be withdrawn (personal communication Margot Jackson).

Over the last few years the ease with which video can be handled on computers has increased considerably. On the other hand bandwidth and PC limitations for the Internet mean that, although diminishing, some Internet users may find accessing this through the Internet has problems. Kiosks with handsets ‘playing’ short health information videos either in English or other languages may have a role. Hahn *et al.* [126–128] developed a talking touchscreen to provide a quality of life assessment for patients with varying literacy skills and computer experience. One item at a time is presented on the computer touchscreen, accompanied by a recorded reading of the question. Various colours, fonts and graphic images are used to enhance visibility, and a small picture icon appears near each text element allowing patients to replay the sound as many times as they wish. Evaluation questions are presented to assess patient burden and preferences. The advantages of ‘talking head presentations’ include: (1) no need for English literacy (the talking head can instruct to press a coloured ‘button’ to continue or go back), (2) improved privacy for users in situations where this is of concern (other people cannot know about what the head is talking). Work is underway on speech recognition for kiosks [129] and ‘intelligent’ kiosks [130], while others have attempted to reduce the amount of text [131].

If evaluation methods involve reading or answering printed questions the needs of illiterate or foreign language minorities may be overlooked; both kiosks and their evaluation methods need to address this [132]. There is of course a body of literature on human computer interface design associated with kiosks (e.g. [133]).

### 3.8. Which Kiosks Are Successful?

Asking ‘How should we evaluate kiosks, booths, and touchscreens?’ is rather like asking how we should evaluate medication; it all depends on the aim of the kiosk (medication). The simple counting of users of opportunistic kiosks is necessary but far from sufficient in being able to evaluate their worth. In 1996 we discussed a series of evaluation studies on the opportunistic kiosk Healthpoint [117]. We asked users for a perceived value in comparison with what the local health board was spending on the provision of leaflets. The average response was 20p per use. There was an average of 60 kiosk usages and 116 users (most usages were by more than one person) per day so that the capital cost of that particular kiosk (£4,000) was ‘paid for’ in one year. Obviously more expensive kiosks or kiosks with less use would be unlikely to be seen as worthwhile. In the 2001 study of NHS kiosks [75] the average daily use was only 12 times a day. The most used kiosk was only used 49 times and a fifth of the 136 kiosks were used less than four times a day. The kiosks were more expensive to buy and to maintain than Healthpoint so it was unsurprising that they were relatively soon decommissioned.

Health commissioners may think that bringing health information to underserved groups is worth more than 20p per use. But some simple arithmetic on any installation will help either make a decision as to whether cost benefit is achievable and at what level of use. For example, if a kiosk costs £3,000 per year (including maintenance and other overheads, and write off of the asset) then the daily cost might be £10 and commissioners can make a judgement about use versus value.

Touchscreens, kiosks or booths integrated into the procedures of a clinic might have other benefits such as more efficient use of clinic time or quantifiable cost savings (e.g. if one receptionist can deal with more patients). Cost effectiveness studies, possibly based on randomised trials but at the very least comparative before-after studies, should be possible.

**Figure 7.**
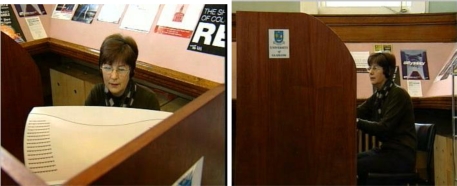
Booth in Whiteinch public library Glasgow (1998) with multimedia touch screen system for cognitive behavioural therapy for stress.

It may well be possible to evaluate the use of booths or touchscreen computers focusing on patient education or treatment, where health outcomes can be defined, in a much more rigorous way. For example, the 2002 NICE review [121] of a number of trials of computerised cognitive behavioural therapy (CCBT) for depression and recommended the use of a stand-alone computer based package in general practice called Beating the Blues, despite a fairly substantial licence cost. Since that time much cheaper (free to the user) Internet based sites have become available for CCBT and trials are underway assessing their cost effectiveness. Most people using such Internet-based CCBT however have access to the Internet at home or work. A study between 1998 and 2000 in Glasgow found that, if referred by their GP, 78% (178/239) patients would use a touchscreen public-library based booth for CCBT (Figure 7) [134].

The Wellpoint kiosk that includes various patient measures could be compared with any occupational nurse time saved, or possibly in terms of a greater take up of a service amongst hard to reach groups.

A great variety of successful uses of kiosks, booths or touchscreens can be found in health promotion where they are used to ‘catch the users eye’ to convey a public health message or used in systems where, for example, patients are referred to use the kiosk or booth as part of patient education. However, clarity of aims and hoped for outcomes of a particular kiosk are essential and documentation of these and how they are to be achieved the best indicator of a successful installation.

Most technology-based services will have a ‘shelf-life’ before needing to evolve or be replaced. A successful kiosk service is one which is seen as achieving its aims during a reasonable life span and which on termination or replacement is thought to have been cost effective. The aims of a kiosk installation might include attitude, use and experience of use, such that a new type of service becomes possible.

### 3.9. Examples of Success (Opportunistic Kiosks)

#### Michigan Health Kiosk:

Internationally the Michigan Health kiosk [78] is often cited, so is it an example of a successful kiosk service? It was first installed about 1998 and was decommissioned in 2004. We do not know the cost of installation and maintenance, but know that there were 100 kiosks and (in 1999) there were approximately 400,000 uses of the kiosks each year. Strecher *et al.* found that users did not differ from non users by ethnicity or gender but that (as it was aimed at stop smoking) it was successful in having more smokers amongst kiosk users than amongst non users. If we make a fairly low cost assumption that the 100 kiosks lasted the six years and that they each had a capital cost of £4000 plus another £4,000 each over six years for maintenance (both hardware/software and programme) and make an optimistic assumption that usage continued undiminished over six years, then the cost per use is £8,000/24,000 = 33 pence. Success could alternatively be measured against other forms of anti-smoking intervention and effectiveness in reduced smoking but attribution of such behaviour change to a single intervention is notoriously difficult. Given the cost of leaflet production (see above) and that it lasted six years I would judge this a successful service, now deceased.

#### Commercial kiosks in the UK:

In Touch with Health and Wellpoint are both commercial organisations so if they manage to keep selling kiosks maybe they can be deemed successful?

Graham Beaumont from Heart of Birmingham had a number of In Touch with Health kiosks. *“Health Exchange is the health and wellbeing support service for the Heart of Birmingham. Information underpins our strategy (based on choosing health) of trusted information, increasingly personalised delivered in places and through people they trust. Kiosks are part of our service (including internet, SMS and now ipTV) designed to ensure that the information that underpins support is validated and accessible. We use kiosks in locations where robustness and privacy are paramount (access areas of libraries, community organisations, primary care centres). They serve 2 purposes in addition to the information: (a) they carry our brand (and therefore our values), (b) they provide a visible focus for our supporters to engage people in a dialogue about their health. We also retrofit kiosks where the technical infrastructure of the organisation is insufficiently robust to support stand-alone pcs and printers. Location and local ownership are key. Location dictates how the service will be used (we know what users are accessing by site) and without local ownership the service collapses (and our credibility is damaged). According to data provided by In Touch with Health there have been a total of 28,080 page views from the Health Exchange kiosks between January 2007 and February 2008. Most of the activity at kiosks is generated by Health Supporters. Our usage differs from Kiosks to internet. Internet usage favours local services and wellbeing. People using our kiosks tend to want to understand their medical conditions. This is a reflection of the age profile of kiosk users (generally older) and the use in community locations by people who have less access to the internet at home (although many users start at the kiosks and then continue searches at home (user feedback survey).”*

**Figure 8.**
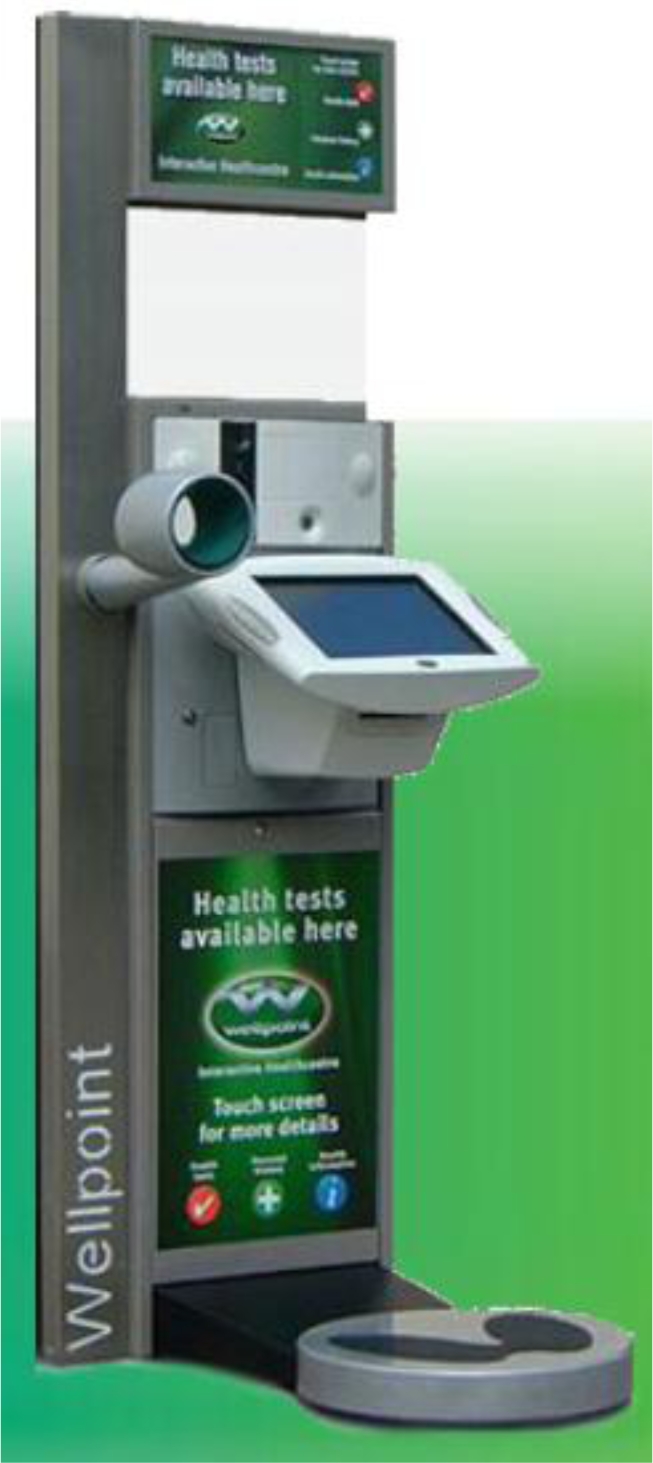
Wellpoint kiosk.

Wellpoint sells their self assessment kiosk (Figure 8) to commercial organisations for occupational health and to retail pharmacy groups. We presume these would not buy the kiosks unless they thought them successful. Chris Dawson (Wellpoint) said “....*many Occupational Health departments don’t want their nurses to do opportunistic screening manually. OH nurses are too valuable a resource. They want to use the Wellpoint kiosk for this so that the OH nurse consultation changes form one of data collection to a consultation about the individuals lifestyle choices and how they can improve their health accordingly...<snip>..’Out-of hours’ screening is becoming more important. If employers offer opportunistic screening they have to offer it to all staff not just staff based in head office and not just to those who can attend within standard working hours (which most OH departments operate to). The energy sector is leading on this type of intervention and we have National Grid, Eon UK and EDF Energy as clients. Their main concerns are offering equal access to all employees to health information and opportunistic screening.*’ An evaluation report from EDF [88] showed 851 uses of the kiosk in 3 months as part of a three month occupational health screening event in 2007. Dawson said ‘*EDF now have 7 Wellpoint units which are rotating around their various sites*’.

#### Other widely available kiosks in UK:

StartHere is a charity that supplies a kiosk not restricted to, but including, health information to health but ‘cover the range of social issues for which an individual might need support across the whole social spectrum including health, housing, education, employment, benefits and welfare issues’. They have a demonstration of a kiosk interface for StartHere East London at http://www.starthere.org/demo/kiosk/Html/index%2002.htm.

#### Opportunistic non health installations:

For opportunistic ‘kiosks’ that aim to gain people’s attention we should consider the applicability of various ‘installations’ such as are found in museums and visitor centres. For example, Dempski *et al.* describe touchable walls [135]. The following examples (provided by HMC Interactive part of the Two Four group) [136] show innovative ways that people can interact with computers, other than simply using a touch screen.

“As you enter the infinity room a giant chocolate bar melts into gloopy puddles beneath you and, when you jump in them, chocolate splashes all over the floor. Then a sprinkling of individual Roses chocolates appear beneath your feet. You won’t believe your eyes when they unwrap as you tread on them — but as you step off they wrap back up. This magical space is controlled by a shock sensitive floor and a series of motion sensors that track you inside the “infinite” space created by a serious of mirrors that make it appear infinitely bigger than it really is.”

“HMC Interactive created cutting edge software for two multimedia exhibits on display with a primary focus on accessibility for all. The museum is the first fully Disability Discrimination Act compliant museum (to open in Britain so central to the challenge was making the software and its interface accessible to as many people as possible. Interactive exhibits allows visitors to explore a series of shop interiors HMC Interactive seamlessly networked four touch–screen tabletops to act as windows on a conveyor belt. Visitors drag items from the belt to their “shopping basket” — triggering the show. Another digital showcase allows visitors to pull items from a virtual display cabinet and manipulate them for a thorough look. The system gives access to films and information covering a range of Wales-wide communities and ideas. HMC Interactive used a completely virtual interface similar to that in the movie Minority Report. Users point at the screen and the computer does the rest. It senses movements as they use their hands to navigate their way through the depths of the exhibit touching everything in virtual reality.”

### 3.10. Examples of Success (Integrated Kiosks)

#### Feedback:

Both In Touch with Health and Opinionmeter kiosks have been assessed by the Picker Institute as ways of capturing patient or public feedback [42,49]. In addition I corresponded with Shirley Dempsey from West Fife who said *“Our Opinionmeters were purchased a number of years back from CRT and are the same as illustrated in the 2007 Picker Report. Our purchase followed on from a recommendation by Fife Health Council [the (then) local NHS watchdog body] that these were a useful tool to elicit user views. These have been utilised occasionally by some of our managed services to gather user opinion at various times, and have been offered on loan to GP Practices to survey opinion. Uptake in the main has been low [needs promoted] .........<snip> but.....<snip> We now use the opinionmeters routinely at our WF annual conferences, and certainly envisage using them more extensively in future at Public Partnership Forum events in the CHP. I certainly think they are a useful tool, and a novel way of eliciting user opinion in a less time-consuming fashion than asking people to complete and return the more traditional [written] questionnaires [‘survey fatigue’]. The analysis is done for you, findings are fairly immediate, opinionmeters are easy to use, attractive to the “techies”, can be set up to award “prizes”, and our experience is that most people don’t need much encouragement to use.”* From the 2007 Picker Report they estimated an annual cost over three years of just over £2,000 for a Standpoint. They were ambivalent in their conclusions in that to get the right people to use it and complete questionnaires may require posters and perhaps staff directing patient to the unit but gave as one of its advantages the fact that it required little minimal staff involvement. Response rates were fairly low. However, if patient feedback is built in to part of a clinical process response rates could be increased.

#### Registration:

Kiosks are being used in patient registration. Here is one U.S. example [112]: Patients can now bypass a receptionist altogether and check in for their appointments by swiping a credit card or driver’s license instead. The new patient check-in kiosk was installed in the autumn of 2007 to help make the check-in process faster. A patient might swipe his credit card, pay part of his bill, receive a receipt, and be successfully checked in for his appointment in under two minutes. About 30 organizations have begun using the kiosk across the country. Killeen Clinic Manager Dean said the Killeen clinic made an excellent trial site for the machine because it sees such a high volume of patients daily. Currently, the kiosk handles the checking-in process for between 50 to 110 patients per day. “It has really helped with wait times,” Dean said. “Some people, who need more service, can talk to receptionists, but for the majority it works really well.” The Killeen clinic has a service representative helping with check-in process at all times, and Dean said many people actually prefer to use the kiosk because it helps protect their confidentiality. “Some people don’t want to talk to other people,” he said. Dean mentioned the “small town gossip” factor that some patients want to avoid when they go to a clinic. People who see someone they know may not want everyone else in the lobby to know why they are seeing a physician, he said. “We want to protect patient confidentiality and the bottom line is that we’re trying to better service our patients.” Steinhardt, too, admitted that while a kiosk helps expedite the process of getting people in to see a physician, there will always need to be people on hand for anyone with special circumstances.

**Figure 10.**
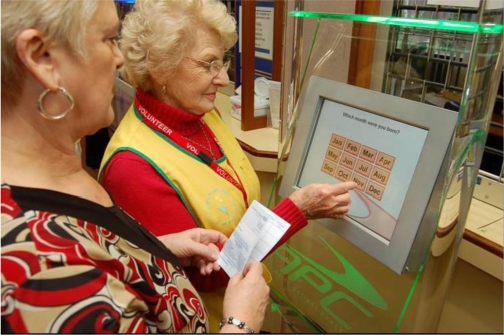
E-reception at Sherwood Forest Hospitals [109].

In the UK, kiosks are starting to be used in hospitals for registration. For example, King’s Mill hospital installed an e-reception system developed by Savience in a new build at King’s Mill Hospital in 2008 [109]. The reception areas deal with around 3,500 patients per week. Patients confirm details using a touchscreen (Figure 10) to book in for outpatient appointment and are directed to waiting area if details are correct or to a ‘rapid changes’ desk to update details if needed. An eWhiteboard tells clinic staff that patient is there and waiting. Clinic staff note on eWhiteboard once patients clinic preparation is complete, so that all clinic staff can see who has arrived and who is ready to continue.

In December 2008, a press report [137] described a similar approach at University Hospitals Birmingham NHS Foundation Trust. Self-service kiosks have been installed in the reception areas of a new hospital build to streamline reception and registration processes, using technology that integrated with its patient administration system. A fully operational kiosk was trialled in Selly Oak hospital for two months, with its supporting system running from the trust’s IT data centre. It proved popular with patients, 51% of whom opted to use it, and improved the efficiency of receptionists. It also improved data quality. If a patient’s details are not correct on the kiosk, they are referred to a receptionist, who can make changes on the core PAS system. The new hospital is due to open in July 2010.

#### Computer-patient interviewing:

In the U.S.A. computer patient interviewing is becoming mainstream (and therefore we can judge successful). Professors Muir Gray and Jeremy Wyatt with Dr Richard Sills organised a workshop in October 2004 at the Institute of Directors [138]. At the conference Professor Gray expressed the view “*I believe that it would be unethical not to do something with this technology and not to do something fast*”. In The U.S.A. developments in this area are now moving quite fast. Quote from Allen Wenner (Primetime Medical Software inc): “*Over 50 Electronic Medical Record system providers now include Instant Medial History (IMH) as part of their software. Of the 20 top selling systems in the U.S., eighteen have deployed IMH, and the other two have committed to implement.’* On the whole my U.S. correspondents tend to assume that patients either complete patient interviews on their home computers or in a practice computer but perhaps using a mouse. Nevertheless this could be via a kiosk to ensure accessibility. Dr John Bachman said (email 4/3/08) *“We have opted for computers in the lobbies and have several in each waiting room It functions as a kiosk and has a front sheet where patients can get education, go online for e-mail, and link to our websites. Jefferson City certainly is an example of a small lobby that uses kiosks.”* Matthew Ferrante [ ferrante@medicalhistory.com] from IMH emailed that “*Galvanon (www.galvanon.com) are finishing production releases of the check in kiosk with IMH this summer. Using IMH at a kiosk by itself (no checkin), here is a link from the American Academy of Family Physicians ‘Practice Transformation” project* http://www.transformed.com/Self-Directed-FirstYear.cfm”.

### 3.11. Why Do Kiosks Fail?

The main barrier to successful long term kiosk use seems to be the way they are bought, maintained (or not), and how benefit is assessed. For example, in the years when hospitals were becoming Trusts many bought Healthpoints for their entrance, often on ‘end of year budgets’ without a clear objective for their use. What is essentially bad financial practice ends up as poor kiosk use. Many kiosks have been part of a research project and funding has ended at the end of the project. For example...”*.... we also had 5 touchscreen computers in pharmacies running <system name>. We had quite a lot of problems with these - mainly with the printers running out of paper and the machine crashing when the print queue got too big. We changed systems to one produced by <company> who were working with <Council> to put touchscreens on the streets. These have been OK and we put a couple in the waiting rooms of GP surgeries. Our problem now is money for maintenance and as bits wear out we have been withdrawing the touchscreens from use.”*

The same has been true of patient interviewing systems as it has for kiosks providing information. A 1997 update on a review of computer-patient interviewing, submitted to the NHS ME [139] concluded “.*..few of the systems reported in the literature have survived as operational systems. Systems developed so far, need to be maintained, often by the person who developed them. When this enthusiast moves on, or develops other interests, the system dies. Ways need to be found of: (i) creating plug and play patient software which requires little maintenance, (ii) ‘embedding’ such systems in routine practice, (iii) creating ‘value-added’ systems, (iv) making this approach more widely known to clinicians and managers.”*

On the other hand commercial kiosks are used routinely for non-health applications (such train tickets) as well as health related. For example, news on the web from Oregon [140] claims that kiosks from Healthnote ‘a provider of healthy-living retail marketing solutions’ are giving a 677% return on investment. It claimed: “c*omplete payback of purchasing.... was obtained after five months of usage and a 677 percent ROI achieved after three years.”* So, kiosks will fail if ownership, responsibility, and potential benefit is not clear.

### 3.12. Equity and the Digital Divide

Although U.K. home Internet access is now over 65% this varies considerably by age, income, and region. However, many studies have shown people obtain health information from another person and, for example, older people may get younger family members to search for them on the Internet. In addition, a 2004, evaluation of a Public Internet Access Points scheme in Scotland reported that 95% of people in urban areas were within one mile of public Internet access and 90% of people in rural areas within five miles [141]. When people actively seek health information (*information pull*) the Internet as a family resource is becoming more accessible. Kiosks probably add little to this availability and other ways of tackling the digital divide may be more effective. These may include better use of libraries. Libraries are already being used in mental health as part of a ‘book referral scheme’, use of ‘gofers’, making Internet easier and cheaper through integration with TV and phone, and getting the software/hardware companies to cater more for the expanding older market. For example, more than 20 (mental health) self-help book schemes have been established across the UK [142]. Within this model, clients presenting to their G.P. with a mild to moderate mental health problem are ‘prescribed’ a suitable self-help title from a standardised list covering many of the mental health difficulties commonly encountered in primary care. The book prescription is then taken to the local public library where all the self-help books are stocked, and clients issued with the specified book in a manner similar to a standard library.

Of course, as cohorts age the ‘age divide’ on Internet use will tend to diminish (e.g. in twenty years time Internet use for all ages is likely to approach 100%; when current 50 year olds become 70 years old their use of Internet will not be less and is likely to be more). On the other hand there is likely to be some new technology divide as the pace of technological change continues [143].

Gilmour in a review of the digital divide argues for the provision of free services at strategic sites and improving the readability and cultural acceptability of health information. Individually focused interventions involve skill development to enable effective navigation of Internet sites, identification of patient and families’ information needs and support to develop evaluation skills. The effectiveness of these interventions in reducing disparities is reliant on nurses and other health professionals’ expertise in accessing, evaluating and using Internet health information in their clinical practise [144].

The digital divide is also of concern in the U.S.A. The American Medical Informatics Association 2003 Spring Congress entitled ‘Bridging the Digital Divide: Informatics and Vulnerable Populations’ convened 178 experts including medical informaticians, health care professionals, government leaders, policy makers, researchers, health care industry leaders, consumer advocates, and others specializing in health care provision to underserved populations [145]. The primary objective of this working congress was to develop a framework for a national agenda in information and communication technology to enhance the health and health care of underserved populations and it resulted in a paper with a number of recommendations. They noted that information and communication technologies, if well designed, can help the underserved more than other groups. They cited the Gustafson’s Comprehensive Health Enhancement Support System (CHESS) [146–149] but noted that such examples were few. The group produced some recommendations for policy, funding, research, and education and training. These had four key themes: revision in payment and reimbursement policies, integration of health care standards, partnerships as the key to success, and broad dissemination of findings including specific feedback to target populations and other key stakeholders [145].

### 3.13. How Current Trends May Influence Kiosks

If we see the integration of TV and web in the home and a more ubiquitous Internet access then kiosks will be used even more as (a) integral to a service or (b) to catch people’s interest (even though those people may have Internet access) rather than a substitute for Internet access. Therefore they will become more local, i.e. have something special and tailored to their actual site.

### 3.14. Videofeeds from Existing Websites and Organisations

Dipex (www.dipex.org.uk) has interviewed people and produced a well used Internet resource of patient experiences. Such videoclips would make an invaluable resource for a kiosk if presented in a kiosk friendly manner. Many other sites such as Age Concern, Cancerbackup, diabetes UK etc have and maintain valuable web sites. However, as they stand these sites are not ‘kiosk friendly’. They cannot be operated by touchscreen and information is often embedded at fairly deep levels or requires searching. This information could be re-used as kiosk content, and in a way which could grab people’s interest if re-formatted into a larger, flatter, more multimedia, kiosk format and were presented showing different content each day or even changing content several times a day by making random or purposeful selections of web content. For example, one could imagine a kiosk in a hospital that presented a random selection of nine different patient experiences from DIPEX and by touching on the person the kiosk use heard that ‘story’. When finished a different random selection of nine people would be showing as the kiosk interface. (A purposeful selection might be made for a particular outpatient clinic). NHS Choices may have a role in encouraging existing website owners to provide ‘kiosk feeds’ from their websites.

### 3.15. Kiosk in Community Development

Various projects have tried to involve members of the community in the development and use of kiosks. For example, I and colleagues involved schools in the early 90s with Healthpoint. Children from a school reviewed the content of Healthpoint and developed their own topics and screens to add to the system [150]. By involving schools or community groups in the content there will be greater ownership and local people will ‘spread the word’ about the utility of the kiosk. NHS Choices could consider a process whereby locally developed content goes through a ‘light’ editorial process before being sent back to the local kiosk. Content thought suitable for national presentation could be included on the web site or via other kiosks. Kiosk content could be made available to all approved companies/organisations in the field (e.g. In Touch, Wellpoint, Starthere etc). Kiosks are being used for patient feedback but they could also be used to capture videoblogs to supplement feedback on NHS Choices website and to be available for replay on an individual kiosk.

### 3.16. Taking Kiosk Development Forward

Kiosks are most likely to work if they are owned and ‘loved’ by people where they are sited, if they integrate in some way with the work flows and processes at that location, and if they are novel and attention grabbing. There are existing companies such as In Touch and Wellness that have found and continue to serve a health kiosk market. On the other hand more cost effective solutions may be available from ‘grass roots’ developments. For kiosks to have novelty value they should not be ‘corporate’ or give the impression that they all present the same information. It would seem to be a mistake for NHS Choices to think of developing its own corporate kiosk. On the other hand, local groups, charities, and others are likely to have innovative ideas for how kiosks could be used and perhaps NHS Choices might issue a call for proposals. It was fairly difficult to find examples of successful kiosk use and it is clear that, despite for example the extensive research carried out by Nicholas *et al.*, or the history of different computer use by patients and the public over the years many of the lessons learned are not well disseminated. Therefore, if a further programme of kiosk development and evaluation were to be considered it should have a strong element of sharing and dissemination of best practice.

Proposals are likely to be successful if they:

- Show they are aware of successes and failures in kiosk use.
- Have a clear statement of how success for the new kiosk would be measured.
- Involve partnership between information, location, and system providers and a plan for how kiosk use will continue beyond the pilot stage.
- Have novel ideas for presentation and integration into location activities.
- Include some independent method for audit of information quality and assessment of cost effectiveness and equity.

Some examples (hopefully applicants would produce more innovative ideas than these):

- Schools in a region might work with national or local charities to develop kiosks with quizzes or ‘local magazines’ possibly re-using web-based materials where appropriate but adding a local and fun dimension to the interaction. Kiosks might be sited in locations targeted at older people. Author-schoolchildren would demonstrate the kiosks to older people and engage them with their use.
- As described above DIPEX might be ‘kiosked’, i.e. presented in a simpler format suitable for touchscreen access. A changing and random selection of nine people talking about a particular topic (e.g. cancer) might be presented in a cancer centre. Nursing or other staff would need to be involved and to encourage patients and their companions to use the kiosks. Success would be measured by patient and nursing opinions and level of use.
- Ethnic minority groups may work with PALS locally, Trusts, charities etc to produce audiovisual interviewing kiosks to collect signs and symptoms using spoken language, or to produce tailored information.
- Kiosks might be used to streamline registration and patient flow in a clinic.
- Kiosk enquiry service in hospital, with a human interface – i.e. telehelp where kiosks at entrance link to one enquiry desk and provide other information.
- Some pilot experiments combining patient interviewing such as provided by IMH together with patient assessment systems such as Wellbeing, and patient/consumer education.

### 3.17. Dos and Don’ts of Health Kiosks

These ‘do’s’ and ‘don’ts’ are about ‘process’, i.e. making your kiosk work to do what you aimed to do. They do not take into account overall aim and cost effectiveness of the kiosk approach. Reference should be made to Nicholas *et al.* [15] who have synthesised their numerous studies into kiosks, web sites and digital TV and providing a ‘handbook’ that contains many ‘do’s’ and ‘don’ts’.

- **DO** involve the staff or other ‘community’ where the kiosk is to be sited. Unless there is buy in at a local level and people are prepared to look after it and make its use effective it will not work. Locally people need to be clear what constitutes successful kiosk use. Can this be expressed as (e.g.) *everyone or a proportion of visitors to that site using the kiosk*, or can it be expressed by some *change of behaviour*, or some *improved data collection* or *patient/public satisfaction*, or by some *cost saving* from using the kiosk to replace some other resource?
- **DON’T** ‘parachute in’ a kiosk if local staff have not been involved in bidding for one. Nicholas *et al.* conclude *‘Kiosks appear to have had little impact on the work of health professionals and reception and managerial staff were found to be inconvenienced by their introduction. Little thought was given by staff to the upkeep of the kiosks when they were purchased. Replenishing paper, trying to fix paper jams, and staying at work late to wait for technicians all created much ill-feeling among practice managers and receptionists.... Locations where a health professional helped patients to use the kiosk had a higher number of users per hour.’*
- If sited in a health service setting, **DO** integrate into clinical practice. Use it for booking in, or for a pre-consultation interview, or for a post-consultation information prescription. However, Nicholas noted from their studies ‘*The ‘patient information prescription’ pads (an attempt to integrate kiosks in surgery routines) were virtually unused, and there was little evidence of doctors referring patients to the system or searching it with them. .... Nurses were more proactive than GPs, and evidence was found to suggest that they valued information as an important part of a patient’s consultation and recovery programme.’* This indicates that integration into clinical practice will not be easy unless clinical staff can see obvious advantages.
- If possible, for opportunistic kiosks, **DO** involve local schools or groups in tailoring the information so that they have ownership and they bring friends and relatives to come to see ‘their work [150].
- **DON’T** include a printer on a kiosk unless someone (as in a bank or an airport) is prepared for a high maintenance job in keeping it working. It will work, as in a bank, if the whole process is cost effective. If production of a booklet or a record or something to be used in the clinical process is the main outcome (see e.g. the U.S. breast screening project [45] then it may be worth it.
- **DO** make the kiosk interesting and highly visible. Particularly if you want opportunistic use the kiosk needs to be clearly visible and people should be curious as to what it does. Healthpoint when it was launched in the early 1990s was new and novel. NHS kiosk when it was launched in 2001 looked too ‘corporate’. (See new and novel interfaces)
- **DON’T** overestimate the need for privacy and do not hide the kiosk away. (Some of the NHS kiosks were in pharmacy back rooms only available by appointment).
- On the other hand, **DON’T** make it look too much like a children’s toy or locate it in a way that this perception is reinforced, otherwise its use may be dominated by toddlers and small children.
- **DON’T** think you are going to solve the digital divide just by providing kiosks. There are other ways of tackling this, for example, by the provision of Internet connected computers in libraries or opening up school computer labs for parents and grandparents, by aiming to bridge the generation gap through projects such as Liverpool scheme on mobile phones[151]. On the other hand, kiosks may help ensure that at least some information is more widely accessible.
- **DON’T** try to replicate the Internet on a kiosk. Standing kiosk use is likely to be for a short period and more focused game or ‘page turning’ applications to capture interest for a short period are needed rather than offering ability to search a database or the web. Seated booth use may be longer.
- If the kiosk just provides opportunistic information **DO** make it clear that information on the kiosk will change frequently otherwise no-one will come to it for repeat use.
- **DO** talk to private sector suppliers
- **DON’T** forget that the TV and other mass media may be the most effective way of getting across a specific health promotion message. Doing so within the context of fiction and celebrity may be as effective as through health promotion video/film. For example, cervical cancer screening rates soared after a SOAP character contracted and died from cervical cancer [30]. Celebrity cancers such as Kylie Minogue can raise awareness much more cost effectively than a 1000 kiosks [152,153]. However, kiosks can be used to personalise, tailor, and reinforce a mass media message.
- **DO** think about using kiosk with sound output and sound assisted input for groups who do not read English.

## 4. Conclusions

Kiosks, booths and other public access computers continue to have a role in 2008 but not as a replica for the Internet. Inequity in Internet access should be addressed directly through economic policy, training and support, through public (library and other) supported access to the Internet (not just health) and indirectly through ‘agents’ helping with ‘health information pull’.

However kiosks, booths, and other public access still have a role in two situations:

- Integrated kiosks: when information provision can be integrated with services, for example, in walk-in centres, outpatient areas, occupational health settings, etc and can focus on a particular task, such as signing in to a service, collection of data (including perhaps physical monitoring) or structured interview, in information prescription, planned education, or providing patient access to their records.
- Opportunistic kiosks: when they can be used in novel and entertaining ways to grab people’s attention and complement other media in health promotion amongst casual users, in both health service and community settings. The basic nature of an opportunistic ‘stand up’ kiosk should not be forgotten – people will use it for maybe two minutes (so there is little point in having deep nested information) and will probably only use it again if they think there is something new. Opportunistic kiosks should be obvious and in areas where there is a large flow of people. The need for privacy can be overstated and depends on the type of site.

The main criteria for kiosk use in any situation is that (a) personnel at the kiosk site have been involved in either bidding for the kiosk or have some motivation to ‘look after it’, and (b) that there are explicitly stated goals that can be used to measure success. These may be simply in numbers of users of certain types, in more effective processes or patient flows, or (in the case of health booths targeted on patient education or therapy) health outcomes.

Interestingly, however, it was not easy to identify numerous examples of current good practice in kiosk use. One of the problems seems to be in the way that kiosks may be bought, commissioned or trialled. Many of the formal evaluation studies in which kiosks, booths, or touch screens have been used have been through research projects funded on short term grants and have not survived into routine use. Some of the systems bought by Trusts or other organisations have not been formally evaluated so their success relies on personal opinion.

Kiosk use can help in improving public health information, patient information and health services. If NHS Choices were to fund further work in kiosks it would best be taken forward through a programme of pilots that included a strong element of sharing and dissemination of best practice.

## Figures and Tables

**Figure 9.**
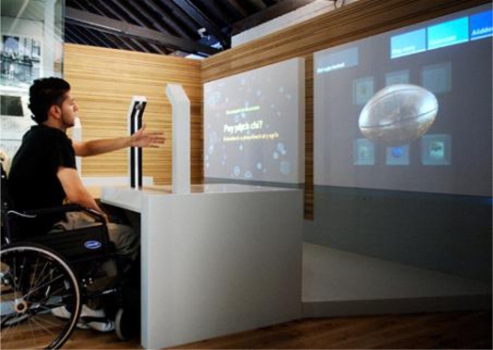
Display at National Waterfront Museum.

**Table 1.** Examples of kiosk use in publications 2002–2008 shown in reverse date order.

**Setting, (Number of kiosks, reference**	**Year publication, country, (type of access)**	**Comments**
Scottish telepresence project (N = ?1) [39]	2008, Scotland, (R)	Newspaper article about teleconsultation where patient booth. Booth includes stethoscope, blood-pressure cuff and thermometer, works on a standard network & needs about 3.5 megabits per second.
Part of cluster RCT in 16 hospitals (so N = 8 intervention). [40]	2007 USA, (O).	Package of interventions to improve antibiotic use in acute respiratory infection: clinical lead, posters, brochures, interactive tailored video kiosk. Modest decrease in antibiotics: but no reporting of kiosk as component.
Kiosks in library, government office, and a McDonald’s in low-income urban locations in Seattle Mar to Oct 2005. (N = 3) [41]	2007 USA, (O)	Users entered child age, were shown selected info. McDonald’s most popular. 28% responded exit survey. 48% had less than high school education, 26% had never used the Internet.
Picker Institute study of patient feedback on two wards in hospitals in Slough. (N = 2) [42]	2007 UK, (R/O)	Two inpatient wards (surgery/urology and respiratory)
Kiosks in aboriginal areas. (N = 11)	2007 Australia, (O)	To improve health literacy in diabetes, alcohol use and child health for remote indigenous populations in Queensland.
Orthopaedic outpatients. (N = 1) [43]	2007 England, (R)	To collect ‘outcome scores’ Oswestry Disability Score from patients
Chicago emergency department [44]	2007 USA, (R)	To promote child safety. Received tailored report
Different sites in metropolitan St. Louis, Missouri, between June 2, 2003, and October 21, 2004. (four kiosks hosted at N = 40) [45]	2006 USA, (O)	*Reflections of You* kiosk. Tailored magazines about breast cancer and mammography. Questions on touch-screen used to generate and print each tailored magazine. 44/110 potential hosts 44 agreed. 7/day valid usages.
Outpatient clinic California (one kiosk, small patient numbers). [46]	2006, USA, (R)	Small scale patient education kiosk for management of uncomplicated urinary tract infections. When published162 women have accessed computer directed therapy.
Primary care waiting room USA. Tailored info. for parents (mean age 26). (N = 1) [47]	2005, USA, (R)	Household safety. Information tailored to child and parent.
Health centres and libraries in deprived areas of Leicester, Sheffield, Nottingham (England) (N = 3) [48]	2005 England, (O)	Written and spoken information on 10 topics in Chinese, Bengali, Gujarati, Urdu, and Mirpuri Punjabi. 2,456 users of 3 kiosks over 10 months.
Outpatient waiting areas. (N = 2) [49]	2005 UK, (R/O)	Patient feedback in outpatient setting (diabetes and orthopaedics clinics) in Edinburgh
Five diabetes clinics in Chicago	2005 USA, (R)	Aimed at low health literacy patients. Relatively less use of the computer among these participants
Emergency departments in USA (N = 1) [50–53]	2004–6 USA, (R)	Used to collect medication information about asthma and make recommendations. Could be used sitting or standing.
Hospital paediatric waiting room in New Mexico USA for Navajo parents (N = 1).[54]	2005 USA, (R)	Aim to improve knowledge of fever management, dental care, sleep position, nutrition, and car seat use
Patient waiting area of multi-specialty clinics, USA (N = 2) [55,56]	2004–6 USA, (O)	Information about eye disease in Spanish and English. Two kiosks for 2.5 years, 1 for 1.5 years. 38,868 user sessions.
(1) kiosk in shopping centre; (2) kiosks in 18 community settings in New South Wales, Australia [57]	2004 Australia, (O)	(1)Three-quarters noticed kiosk and 21% used it. (2)57064 user sessions, i.e. 19 user sessions on average/day
Primary care waiting room near Edinburgh [58]	2004 Scotland (O)	Studied characteristics of users Vs non users in a postal survey of just under 200 patients
20 In Touch with Health kiosks sited in UK primary care [59]	2003 UK, (O)	Studied 20 kiosks over three years and half years. Novelty value for 4–5 months followed by decline
Kiosks sited in churches, senior centres, schools, shopping malls, grocery stores, hospitals (N = 100) [60]	2003 USA, (O)	Addition of Alzheimer ‘channel’ for Michigan Kiosk project. 100 kiosks sited in seniors centres, shopping malls etc.
In Touch and NHS kiosk compared with Surgery Door web site. England [61]	2003 UK, (O)	Comparison of log files (time spent etc) between web information and kiosk information
Nutrition education in food assistance programs among Hispanics in USA [62]	2002 USA, (R)	Bilingual Spanish-English. Comparative cost-effectiveness study Vs peer educators
Outpatient waiting room, diabetes eye examinations. [63,64]	2002, USA, (R)	Aimed at underserved populations
NHS Direct kiosks England [65]	2002 UK, (O)	Comparison of one month’s log data between 120 kiosks
Patient interviewing for anxiety and depression. [66]	2002 USA, (R)	Validation study of computerised HADS versus paper HADS (N = 1,304)

Key to type of use: R = referred or invited; O = Opportunistic

**Table 2.** Bigger installations of health kiosks.

Country	Kiosk name	Max number approx	Dates	Sources
UK	Healthpoint	60	1989–1998	[31] (Jones, personal knowledge)
UK	NHS Kiosk	136	Sep 2000-c.2005	[75] (Bob Gann email)
UK	In Touch with Health	200	Approx 1997-Continuing	[67] emails from Kevin Snowball (In Touch with Health)
USA	Michigan	100	1998–2004	[60,78,113]
Aust	Health CHIPS	20	Main tranch of kiosks no longer supported, used in certain niche ‘markets’	[57] (email Trevor Hazell) [114,115]
UK	Wellpoint	268	2003-Continuing	Emails and phone calls Chris Dawson (Wellpoint)
UK	StartHere BT Street Kiosks Colorama –iStop Community projects	130 50 55	2004–2007 2007–2008 2000-continuing	Emails from Mark Worger, Business Development Officer StartHere
UK	Elephant kiosks	164	Current installations in Staffordshire and Suffolk Primary Care and, Cambridgeshire Hospital	Email Mark Worger on behalf of Annette Walker (Elephant Kiosks)
